# Unsalable Vegetables Ensiled With Sorghum Promote Heterofermentative Lactic Acid Bacteria and Improve *in vitro* Rumen Fermentation

**DOI:** 10.3389/fmicb.2022.835913

**Published:** 2022-05-12

**Authors:** Daniel L. Forwood, Devin B. Holman, Alex V. Chaves, Sarah J. Meale

**Affiliations:** ^1^School of Agriculture and Food Sciences, Faculty of Science, The University of Queensland, Gatton, QLD, Australia; ^2^Agriculture and Agri-Food Canada, Lacombe Research and Development Centre, Lacombe, AB, Canada; ^3^School of Life and Environmental Sciences, Faculty of Science, The University of Sydney, Camperdown, NSW, Australia

**Keywords:** 16S rRNA sequencing, unsalable vegetable silage, *in vitro* rumen fermentation, microbial profiling, sorghum

## Abstract

This study characterized the nutritive and microbial profiles and the fermentation characteristics of silage with the following compositions on a dry matter (DM) basis: (1) 100% sorghum, (2) 70% sorghum + 30% carrot or pumpkin, and (3) 40% sorghum + 60% carrot or pumpkin. The treatments were further divided based on the addition or no addition of a probiotic inoculant. After 70 days of ensiling, the silage was incubated for 48 h using the *in vitro* batch culture technique. Crude protein and non-fiber carbohydrates in the silage increased (*P* ≤ 0.01) by 5.7 percent point (pp) and 9.6 pp, respectively, with pumpkin at 60% DM. The V4 region of the 16S rRNA gene was sequenced to profile pre-ensiled and ensiled archeal and bacterial communities. Silages containing carrot or pumpkin strongly influenced the microbial structure (PERMANOVA: *R*^2^ = 0.75; *P* < 0.001), despite the ensiled treatments being dominated by *Lactobacillus* spp., except for the control, which was dominated by *Weissella* and *Pediococcus* spp. (*P* < 0.01). Linear discriminant analysis indicated that carrot and pumpkin silages were responsible for the increased relative abundance of *Lactobacillus* and *Acinetobacter* spp. (log LDA score ≥ 2), respectively. After 48 h of incubation, carrot and pumpkin inclusion increased (*P* < 0.01) the *in vitro* DM digestibility by 22.5 and 31.3%, increased the total volatile fatty acids (VFAs) by 16 and 20.6% (*P* < 0.01), respectively, and showed a tendency (*P* = 0.07) to increase the gas production. Therefore, this study supports the use of carrot or pumpkin in sorghum silages to maximize feed digestibility and total VFA concentrations.

## Introduction

Ensiling is a method of feed preservation facilitated by anaerobic lactic acid bacteria through the fermentation of water-soluble carbohydrates within the plant material. Vegetable feedstuffs, such as carrots and pumpkins, contain 89–94% moisture (Zinash and Woldetsadik, 2013; Bender et al., 2020), neutral pH, and high water activity (Tournas, 2005), thus increasing their susceptibility to microbial spoilage (Pahlow et al., 2003). To mitigate this, the inclusion of a fibrous substrate, such as sorghum (Wadhwa et al., 2013), can promote a more acidic ensiling environment (McDonald et al., 1991), thus inhibiting clostridial growth in silage. Although sorghum is low in crude protein, legumes are typically utilized to improve the feeding value of the resultant silages (Titterton and Maasdorp, 1997). Vegetables such as carrot or pumpkin at 20 or 40% dry matter (DM) are potential candidates for ensiling and for use as fodder substitutes for sorghum or maize. Silages supplemented with vegetables favor the growth of *Lactobacillus* spp., simultaneously increasing the microbial diversity with sorghum, while maintaining *in vitro* fermentation and gas parameters [mL/g DM gas; percent, mg/g DM; mg/g CH_4_ digestible dry matter (DMD)] with maize (Forwood et al., 2019; Hooker et al., 2019). Moreover, *in vitro* studies have also utilized pumpkin ensiled at 30% DM with corn stover, which increased *in vitro* DM digestibility by 25%, while consequently decreasing gas production by 6% (Crosby-Galván et al., 2018).

Inoculation of plant material with probiotics containing lactic acid bacteria prior to the ensiling process has previously increased lactic acid production, thus improving silage fermentation (Weinberg et al., 2004). Increased lactic acid production may reduce aerobic stability through the utilization of lactic acid by yeasts and molds (Puntillo et al., 2020), despite increasing the *in vitro* DM and organic matter (OM) digestibility of wheat straw-concentrate silage (Malik and Sharma, 1998). Of the lactic acid-producing bacteria, *Lactiplantibacillus plantarum* and *Pediococcus* spp., have been utilized as probiotic inoculants in sorghum silage production, which improve silage quality through rapid pH decline and increased lactic acid production, consequently reducing the growth of undesirable spoilage microbes during the ensiling process (Alhaag et al., 2019).

This study aimed to characterize silage production parameters, nutrient composition, and microbial communities colonizing unsalable carrot or pumpkin silages at 0, 30, or 60% DM, with or without a probiotic inoculant. Further, the study also aimed to analyze the silage for their influence on *in vitro* fermentation, including gas and CH_4_ production, silage digestibility, and total volatile fatty acid (VFA) concentration. This study hypothesized that increasing the proportion of carrot or pumpkin to 60% DM would result in silage of a greater fermentation quality dominated by *Lactobacillus* spp. Consequently, it is also expected that *in vitro* DM digestibility will be improved with an increase in the amounts of carrots or pumpkins.

## Materials and Methods

### Silage Production

Sorghum (*Sorghum bicolor* L. Moench) was collected during harvest from the University of Queensland (Gatton, QLD 27°56′ S, 152°28′ E) in January 2019. Carrots and pumpkins deemed unsuitable for human consumption (considered unsalable) were collected from Kalfresh Pty Ltd. (Kalbar, QLD, Australia). The following treatments (*n* = 10) were included on a DM basis: (1) 100% sorghum (control), (2) 70% sorghum + 30% carrot or pumpkin, and (3) 40% sorghum + 60% carrot or pumpkin, with or without a commercial probiotic (BioSoil Probiotics, OzProbiotics, Penrith, NSW, Australia). The probiotic bacteria used for inoculation were as follows: *Bacillus subtilis, Bifidobacterium animalis, Bifidobacterium bifidum, Bifidobacterium longum, Enterococcus lactis, Streptococcus thermophilus, Lactobacillus acidophilus, Lactobacillus delbrueckii* subsp. *bulgaricus, Lactobacillus casei, Limosilactobacillus fermentum, L. plantarum*, or *Saccharomyces cerevisiae*. Prior to the ensiling process, approximately 3 mL of probiotics were applied to the candidate material prior to packing into PVC mini silos (*n* = 40; diameter 90 mm × height 55 cm × volume 3,500 cm^3^; 5 kg material capacity), with the probiotic solution and silo packing conducted according to the methods proposed by Forwood et al. (2019).

After 70 days of ensiling, mini silos were weighed and opened. The top 10 cm of each mini silo was considered to be spoiled and was discarded. Silage designated for the analysis of DM was collected in duplicate *via* the methods described by Forwood et al. (2019) and dried in an oven at 65^°^C until a constant weight was obtained. Similarly, approximately 15 g of silage was obtained from each mini silo and processed for analysis of pH, VFA, and organic acids, while a 70 g duplicate sample from each mini silo was collected for DNA extraction and 16S rRNA gene sequencing and stored at −20^°^C until further processing.

### Chemical Composition

Dried silage samples were placed in a furnace at 600°C for 2 h [AOAC (942.05 2002)] for the analysis of ash content. Samples designated for neutral detergent fiber (aNDF), crude fat, and crude protein (CP) analyses were ground and passed through a 1 mm screen, and 0.5 g of the sample was weighed and placed into ANKOM F57 bags (ANKOM Technol. Corp., Fairport, NY, United States). Neutral detergent fiber (aNDF) content was determined according to the methods described by Van Soest et al. (1991) with a modification in using ANKOM 200/220 Fiber Analyzer (ANKOM Technol. Corp., Fairport, NY, United States). Sodium sulfite and amylase, along with residual ash, were added to the aNDF samples for aNDF analyses. The crude fat content of the feed was determined by extraction with ether as described for lipid extraction [AOAC (929.29 1995)] and modified for an ANKOM Fat Analyzer (ANKOM Technol. Corp., Fairport, NY, United States). The nitrogen content of the feed was determined using a LECO CN928 carbon/nitrogen combustion analyzer (Leco, St Joseph, MI 49085, United States; AOAC 990.3). The instrument was set up as per the manufacturer’s recommendations. Briefly, the instrument was standardized with EDTA, combustion temperature was adjusted to 1,100°C, and approximately 0.3 g of ground feed sample was weighed into ceramic boats and analyzed. Crude protein was calculated as mineral nitrogen × 6.25, while non-fiber carbohydrates (NFCs) were calculated as follows:


[100-(%aNDF+%CP+%Fat+%Ash)],

where, %aNDF; %CP; %Fat; %Ash = % on a DM basis.

### pH, Volatile Fatty Acids, and Organic Acids

From each PVC mini silo, 15 g of silage was collected, combined with 135 mL of distilled water, and blended at room temperature. The resulting solution was filtered through a cheesecloth, and 15 mL of the filtrate was collected, mixed, and immediately measured for pH using a Hanna Edge HI2002 pH meter (Hanna Instruments, Woonsocket, RI, United States). Approximately 35 mL of the remaining filtrate was placed on ice prior to centrifugation for 15 min at 10,000 × *g* and cooled to 4^°^C. Metaphosphoric acid [1 mL; 5:1 ratio (v/v)] was combined with 5 mL of filtrate replicates and stored at −20^°^C until the analysis for the content of VFAs and organic acids.

Volatile fatty acids and organic acids were analyzed as per the methods proposed by Playne (1985). Briefly, 1.5 mL of each sample was centrifuged at 12,000 rpm for 2 min. An aliquot of 1.2 mL was combined with 0.2 mL crotonic acid solution, kept at room temperature, and centrifuged for a further 10 min at 12,000 rpm. The supernatant was transferred to an autosampler vial for analysis of VFA by gas chromatography according to the method proposed by Forwood et al. (2019). Concentrations of organic acids and VFA were expressed in mM, and ethanol content was expressed as %.

### *In vitro* Fermentation Characteristics and Determination of Gas and CH_4_ Production

Rumen samples for *in vitro* fermentation and determination of gas and CH_4_ production parameters were obtained from cannulated Holstein steers (*n* = 3) housed at The University of Queensland Gatton Dairy under the guidance and approval of the Animal Ethics Committees of The University of Queensland (approved protocol number: AE35581). The rumen samples were collected as per the methods proposed by Meale et al. (2012), and inoculum for batch culture was prepared *via* the methods proposed by Menke et al. (1979).

*In vitro* batch culture incubation and analysis of gas and CH4 production parameters were conducted according to the methods proposed by Forwood et al. (2019), with some modifications. Briefly, incubation bottles were prewarmed for 1 h in a Ratek OM25 Digital Shaking Incubator (Ratek Instruments Pty Ltd, Boronia, VIC, Australia) maintained at 39^°^C. Further, the bottles were filled, gassed under a stream of CO_2_, sealed, and then subsequently placed into the incubator at 90 rpm for 24 h. Batch culture incubation was repeated three times, with two treatment replicates per run, and included blank samples containing 25 mL of rumen inoculum only.

The production of gas was determined by using a water displacement apparatus (Fedorah and Hrudey, 1983), and pH was determined as per the methods proposed by Meale et al. (2012). Bags were removed from bottles, placed on ice to cease fermentation, thoroughly rinsed with distilled water, and finally placed in an oven at 65^°^C until a constant weight was obtained. Bags were subsequently weighed prior to the calculation of *in vitro* DM digestibility.

### Sequencing of the Archaeal and Bacterial 16S Genes

The V4 hypervariable region of the archaeal and bacterial 16S rRNA gene was amplified and sequenced as previously described by Forwood et al. (2020) using an Illumina MiSeq instrument and the MiSeq Reagent Kit v2 with 500 cycles (Illumina, Inc., San Diego, CA, United States), according to the manufacturer’s instructions. DADA2 v. 1.16.0 (Callahan et al., 2016) in R v. 4.0.2 was used to process and quality-filter all the sequences. The maximum number of expected errors permitted was 2. The forward and reverse reads were trimmed to 201 and 210 bp, respectively, and merged with a minimum overlap of 100 bp, and chimeras were removed. Taxonomy was assigned to these remaining sequences, referred to here as operational taxonomic units (OTUs) at 100% similarity, using the RDP naïve Bayesian classifier and the SILVA SSU database release 138 (Quast et al., 2013). OTUs classified as chloroplasts and mitochondria were removed prior to the analyses.

The number of OTUs per sample (richness), the Shannon diversity index, and the inverse Simpson’s diversity index were calculated in R using vegan 2.5–6 and Phyloseq 1.32.0 (McMurdie and Holmes, 2013). The bacterial community structure was assessed using Bray–Curtis dissimilarities that were calculated with the vegan 2.5-6 package (Oksanen et al., 2019), and the effect of vegetable mixture and probiotic supplementation was determined using a permutational multivariate analysis of variance (PERMANOVA; adonis2 function) within the vegan 2.5–6 package. To account for unequal sequencing depth, all samples were randomly subsampled to 1,400 sequences per sample, prior to the calculation of the diversity measures. However, as there was greater sequencing depth in the ensiled samples, these samples were subsampled to 9,300 sequences to retain as many sequences as possible for calculating the Bray–Curtis dissimilarities. Fermentation parameters were fit to the non-metric multidimensional scaling (NMDS) ordinations of the Bray–Curtis dissimilarities using the envfit function within vegan with 10,000 permutations. Pre-ensiled and ensiled OTUs were visualized for comparison using the heatmap function of ampvis2 v. 2.7.9 on R v. 4.0.2 (Andersen et al., 2018). All the 16S rRNA gene sequences were submitted to the Sequence Read Archive under BioProject PRJNA699618.

### Statistical Analysis

Chemical composition, silage pH, VFAs, organic acids, and alpha diversity measures were analyzed as a completely randomized design using the MIXED procedure of SAS with the fixed effects of vegetable (carrot vs. pumpkin), level (0, 30, and 60% DM), with or without probiotics, and their interactions. Mini silo within treatment was considered the random effect. Further, bacterial genera > 0.1% relative abundance (RA) and those were detected in > 15% samples considered biologically relevant and analyzed by the MIXED procedure of SAS using the aforementioned fixed- and random-effects parameters.

The results of *in vitro* fermentation and the production of VFAs, gas, and CH4 were analyzed as described earlier, but random effects were defined as *in vitro* fermentation run and run × treatment. All results were expressed as LSMEANS with standard error of the mean (SEM), while *P* ≤ 0.05 was considered statistically significant, and tendencies were reported when 0.05 < *P* ≤ 0.10. Experimental units in this study consisted of individual mini silos and incubation run for the *in vitro* fermentation.

Normal distribution of the data was tested using the UNIVARIATE procedure of SAS, while the influence of vegetable type, level, and probiotic of the silage microbial community structure was determined using PERMANOVA (adonis2 function) and Bray–Curtis dissimilarities in R using the vegan 2.5–6 package (Oksanen et al., 2019). The linear discriminant analysis effect size (LEfSe) method^1^ (Segata et al., 2011) was conducted using Galaxy version 1.39.5.0^2^ (Jalili et al., 2020) to determine bacterial diversity between the treatments. Differential abundance was assumed when LDA scores were ≥ 2.0 and *P* ≤ 0.05 (Li et al., 2020).

## Results

### Chemical Composition

Crude protein content in silage DM was the only parameter that was influenced by vegetable × level × probiotic (*P* = 0.03; data not presented). The subsequent results are presented based on the addition or no addition of probiotics: uninoculated (no) or inoculated (yes). The CP content increased (*P* < 0.01; Table 1) in uninoculated silages by 38.1 and 52% with pumpkin at 30 and 60% DM, while the content increased by 14.5 and 22.3% with pumpkin at 30 and 60% DM, respectively. Consequently, the fiber (aNDF) concentration decreased (*P* = 0.02) by up to 22.8% in inoculated silages when vegetables were included, irrespective of the type (Table 1).

**TABLE 1:** Chemical composition of silages with carrot or pumpkin at 0, 30, or 60% DM, with (yes) or without (no) a probiotic inoculant.

No probiotic		Carrot		Pumpkin			*P-*values				
	0	30	60	30	60	SEM	Veg	Level	Veg × Level	Linear	Quadratic
DM content, %	27.3	21.8	14.4	20.1	16.7	1.32	0.87	<0.01	0.38	<0.01	0.69
CP, % DM	9.34	10.4	10.2	12.9b	14.2a	0.36	<0.01	<0.01	<0.01	<0.01	0.03
NFC, % DM	20.4	19.9	24.0	25.0	27.9	3.59	0.35	0.39	0.75	0.19	0.82
EE, % DM	8.27	8.09	5.55	4.87	5.26	0.68	0.08	0.01	0.10	<0.01	0.57
aNDF, % DM	50.1	48.0	46.9	44.8	35.8	2.81	0.08	0.04	0.19	0.02	0.79
Ash, % DM	11.9	13.7	13.4	12.4	15.8	0.83	0.60	0.06	0.18	0.02	0.77

**Yes probiotic**		**Carrot**		**Pumpkin**			***P*-values**				
	**0**	**30**	**60**	**30**	**60**	**SEM**	**Veg**	**Level**	**Veg × Level**	**Linear**	**Quadratic**

DM content, %	26.8	20.8	14.9	19.6	16.8	0.84	0.73	<0.01	0.23	<0.01	0.17
CP, % DM	10.3	10.8	10.8	11.8a	12.6a	0.26	<0.01	<0.01	0.03	<0.01	0.29
NFC, % DM	20.1	24.2	28.6	27.5	29.2	1.61	0.37	<0.01	0.60	<0.01	0.37
EE, % DM	8.61	7.87	7.22	11.7	8.33	1.41	0.20	0.40	0.45	0.57	0.24
aNDF, % DM	49.2a	45.1b	40.6c	38.1b	38.0b	0.94	< 0.01	<0.01	0.02	<0.01	0.02
Ash, % DM	11.8	12.0	12.7	11.0	11.8	0.49	0.15	0.30	0.56	0.36	0.22

*DM, dry matter; CP, crude protein; NFC, non-fiber carbohydrates; EE, ether extract; aNDF, neutral detergent fiber; SEM, standard error of the mean. Letters a–c indicate differences (P ≤ 0.03) between levels within vegetable (i.e., interaction Veg × Level was split by vegetable).*

### Silage pH and Organic Acids

Acetic acid, lactic acid, total VFA, and ethanol concentrations were influenced by vegetable × level × probiotic (*P* ≤ 0.04; data not presented). The results obtained following uninoculated (no) and inoculated (yes) treatments are presented in the following section.

#### No Probiotics

**FIGURE 1:**
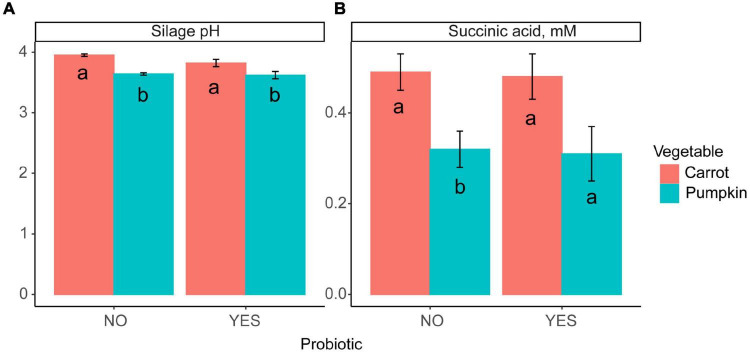
A bar plot of **(A)** silage pH and **(B)** succinic acid concentration by vegetable (*P* ≤ 0.02) inclusion with probiotic use. Letters a,b indicate differences (*P* ≤ 0.04) between the probiotic treatments.

Inclusion of carrot at 60% DM in uninoculated ensiled sorghum silages increased the silage pH and the concentrations of acetic acid, total VFA, and lactic acid, while the ethanol concentration was reduced from 18.9 to 6.27 ± 1.39% DM (*P* ≤ 0.01; Table 2). The fixed effect of vegetable type was significant, where the silage pH was greater (*P* = 0.04; Figure 1A) with carrot, rather than when pumpkin was included.

**TABLE 2:** Fermentation parameters of sorghum combined with carrot or pumpkin at 0, 30, or 60% DM after 70 days of ensiling, split by with probiotic (yes) or without (no).

No probiotic		Carrot		Pumpkin			*P*-values				
	0	30	60	30	60	SEM	Veg	Level	Veg × Level	Linear	Quadratic
Silage pH	3.67c	3.81b	4.35a	3.68	3.55	0.03	<0.01	<0.01	<0.01	<0.01	0.06
Ethanol, % DM	18.9a	6.97b	6.27b	24.6a	18.1b	1.39	<0.01	0.02	<0.01	0.01	0.60
Total VFA, mM	3.90c	13.6b	20.7a	5.42	6.79	1.22	<0.01	<0.01	<0.01	<0.01	0.54
**Organic acids**											
Lactic acid, mM	15.0a	13.5a	2.82b	16.1	16.8	0.73	<0.01	<0.01	<0.01	<0.01	0.01
Succinic acid, mM	0.31	0.50	0.67	0.31	0.35	0.06	0.02	0.05	0.11	0.02	0.90
**Volatile fatty acids**											
Acetic acid, mM	3.56c	13.3b	20.0a	5.18	6.55	1.14	<0.01	<0.01	<0.01	<0.01	0.44
Valeric acid, mM	0.28	0.11	0.08	0.12	0.18	0.12	0.69	0.26	0.87	0.22	0.35

**Yes probiotic**		**Carrot**		**Pumpkin**			***P*-values**				
	**0**	**30**	**60**	**30**	**60**	**SEM**	**Veg**	**Level**	**Veg × Level**	**Linear**	**Quadratic**

Silage pH	3.68	3.95	3.83	3.60	3.59	0.10	0.04	0.60	0.22	0.82	0.37
Ethanol, % DM	21.7a	7.30b	7.20b	8.05b	17.4a	1.41	0.02	<0.01	<0.01	<0.01	<0.01
Total VFA, mM	6.28c	18.3a	10.1b	10.3a	9.14a	0.94	<0.01	<0.01	<0.01	<0.01	<0.01
**Organic acids**											
Lactic acid, mM	14.8	9.41	14.45	14.8	16.7	2.71	0.23	0.42	0.53	0.79	0.21
Succinic acid, mM	0.35	0.54	0.56	0.26	0.32	0.09	0.06	0.61	0.35	0.34	0.96
**Volatile fatty acids**											
Acetic acid, mM	5.51c	17.8a	9.97b	9.89a	9.00a	0.92	<0.01	<0.01	<0.01	<0.01	<0.01
Valeric acid, mM	0.31	0.15	0.10	0.28	0.06	0.10	0.74	0.15	0.75	0.06	0.83

*SEM, standard error of the mean; VFAs, volatile fatty acids; P-values for succinic and valeric acids for Veg × Level × Prob ≥ 0.75. Letters a–c indicate differences (P ≤ 0.05) between levels within vegetable (i.e., interaction Veg × Level was split by vegetable).*

#### Yes Probiotics

Silage pH and lactic, succinic, and valeric acid concentrations were not influenced by vegetable × level in inoculated silages (*P* ≥ 0.11; Table 2 and Figure 1B); however, ethanol, acetic acid, and total VFA concentrations were all influenced by vegetable × level (*P* < 0.01). When pumpkin at 60% DM was included, ethanol concentration was 17.4 ± 1.41%; however, the ethanol content was lower at 7.20 ± 1.41% when carrot was included on a DM basis. Conversely, when carrots were included at 30% DM, acetic acid and total VFA concentrations were greatest at 17.8 vs. 9.89 ± 0.92 and 18.3 vs. 10.3 ± 0.94% DM compared to pumpkin at 30% DM, respectively. Moreover, the effect of the vegetable type was significant, with the silage pH of carrot greater by 5.5% compared to that of pumpkin (*P* < 0.01; Figure 1A).

### Bacterial Community Composition of Pre-ensiled and Ensiled Samples

There were 24,002 ± 2,307 16S rRNA gene sequences per sample after quality filtering and removal of plant (chloroplast) contamination, and 1,076 OTUs were detected among all the 43 samples. Further, the ensiling process reduced the number of detected sequences by 60% (*P* < 0.01). In the pre-ensiled samples, there was an effect (*P* < 0.01; data not presented) of vegetable × level × probiotic on the Shannon diversity index, while there was no effect (*P* ≥ 0.15; data not presented) of the three-way interaction on the ensiled samples. Therefore, the samples were divided into groups based on probiotic addition: probiotic inoculation (yes) or no probiotic inoculation (No). The samples were further analyzed for the effects of vegetable, level, and vegetable × level.

#### Pre-ensiled Samples

All alpha diversity measures (number of OTUs, Shannon’s and inverse Simpson’s diversity indices) in the pre-ensiled samples were influenced (*P* < 0.01; Table 3) by vegetable × level, with the values in all the treatments greater than those of the control, except for pumpkin at 30% DM. The number of OTUs decreased by 12 OTUs from 27 ± 5.93 to 15 ± 5.93 with 0% vegetable addition.

**TABLE 3:** Pre-ensiled communities of sorghum ensiled with carrot or pumpkin at 30 or 60% DM, separated by probiotic.

No probiotic		Carrot			Pumpkin			*P*-values				
	0	30	60	0	30	60	SEM	Veg	Level	Veg × Level	Linear	Quadratic
No. OTUs	27b	69a	78a	27b	15b	63a	5.93	<0.01	<0.01	0.01	<0.01	0.21
Inverse Simpson’s	1.83b	13.2a	13.3a	1.83b	2.16b	8.39a	0.76	<0.01	<0.01	<0.01	<0.01	0.09
Shannon	1.22b	3.18a	3.24a	1.22b	1.10b	2.82a	0.11	<0.01	<0.01	<0.01	<0.01	0.88

**Yes probiotic**		**Carrot**			**Pumpkin**			***P*-values**				
	**0**	**30**	**60**	**0**	**30**	**60**	**SEM**	**Veg**	**Level**	**Veg × Level**	**Linear**	**Quadratic**

No. OTUs	41b	63a	71a	38ab	30b	51a	3.79	<0.01	<0.01	0.02	<0.01	0.25
Inverse Simpson’s	1.77b	11.9a	13.6a	1.83b	5.99a	7.27a	0.98	<0.01	<0.01	0.03	<0.01	0.01
Shannon	1.31b	3.08a	3.23a	1.35c	2.39b	2.63a	0.06	<0.01	<0.01	<0.01	<0.01	<0.01

*OTUs, operational taxonomic units; SEM, standard error of the mean. Letters a and b indicate differences (P ≤ 0.05) between levels within vegetable (i.e., interaction Veg × Level was split by vegetable). Interactions of Prob, Veg × Prob, Level × Prob, and Veg × Level × Prob were significant for the Shannon’s diversity index and number of OTUs (Level × Prob; P = 0.02).*

#### Ensiled Samples

##### No Probiotics

**FIGURE 2:**
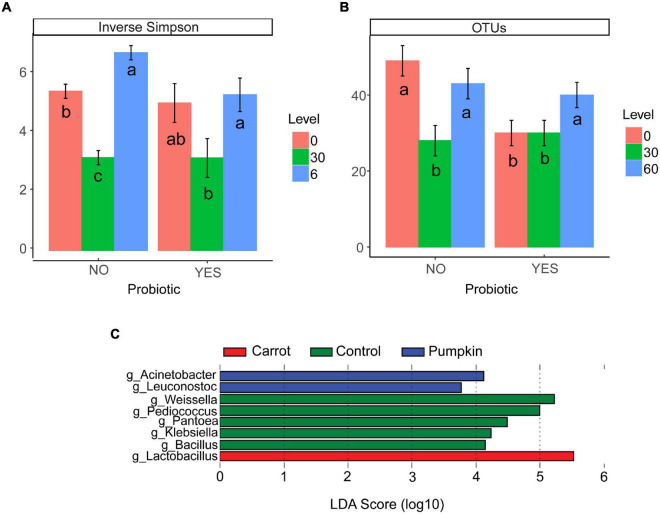
**(A)** Inverse Simpson’s diversity index and **(B)** the number of OTUs in ensiled samples that were influenced by vegetable level (*P* ≤ 0.01) when compared with probiotic use. When split by probiotic use, there was no effect of vegetable type (*P* ≥ 0.14; data not presented). Letters a–c indicate differences within probiotic treatments (*P* ≤ 0.05). Differentially abundant genera **(C)** in the microbiota of sorghum ensiled as a control, or with carrot or pumpkin supplementation for 70 days using the linear discriminant analysis effect size (LEfSe) method.

There was no effect (*P* ≥ 0.15; Table 4) of vegetable × level on the number of OTUs or the inverse Simpson’s diversity index, and only for the Shannon diversity index, it decreased (*P* = 0.05) with carrot at 30% DM. Vegetable levels affected both the number of OTUs and the inverse Simpson’s diversity index of the silage microbiota, where the number of OTUs was greater (*P* < 0.01) with 0 and 60% DM vegetable inclusion in the uninoculated silages (data not presented). Also, the inclusion of vegetable at 30% DM decreased the inverse Simpson’s diversity index by 27%, compared to the inclusion of vegetables at 60% DM (*P* < 0.02; Figure 2A).

**TABLE 4:** Measures of alpha diversity for sorghum ensiled with carrot or pumpkin at 0, 30, or 60% DM for 70 days, separated by probiotic.

No probiotic		Carrot		Pumpkin			*P*-values				
	0	30	60	30	60	SEM	Veg	Level	Veg × Level	L	Q
No. OTUs	49	21	44	36	41	5.56	0.40	0.02	0.28	0.34	0.01
Inverse Simpson’s	5.42	2.68	6.85	3.64	6.61	0.34	0.42	<0.01	0.25	0.01	<0.01
Shannon	2.23a	1.34b	2.57a	1.93	2.39	0.12	0.23	<0.01	0.05	0.10	<0.01

**Yes probiotic**		**Carrot**		**Pumpkin**			***P*-values**				
	**0**	**30**	**60**	**30**	**60**	**SEM**	**Veg**	**Level**	**Veg × Level**	**L**	**Q**

No. OTUs	30	25	40	35	40	3.79	0.14	0.02	0.15	0.01	0.08
Inverse Simpson’s	5.02	3.25	6.05	3.05	4.55	0.83	0.40	0.07	0.58	0.75	0.03
Shannon	2.01	1.77	2.26	1.66	2.02	0.17	0.37	0.09	0.71	0.45	0.04

*OTUs, operational taxonomical units; SEM, standard error of the mean; Letters a and b indicate differences between treatments (P ≤ 0.05) and between levels within the vegetable type (i.e., interaction Veg × Level was split by vegetable). P-values for Shannon’s and inverse Simpson’s diversity indices for interactions Veg × Prob, Level × Prob, and Veg × Level × Prob were ≥ 0.15.*

##### Yes Probiotics

The richness (number of OTUs) of the inoculated silage microbiota was affected by the vegetable level, where greater richness was noticed only with the vegetable at 60% DM (*P* = 0.02; Figure 2B).

### Archaeal and Bacterial Relative Abundance in Pre-ensiled and Ensiled Samples

Prior to the ensiling process, the microbiota composition of the 100% sorghum and 30% pumpkin treatments was dominated by the members of the *Pantoea* genus (76.5 ± 6.09% RA; *P* < 0.01; Supplementary Table 1), irrespective of the addition of the probiotic. In contrast, the pre-ensiling material containing carrot at 60% DM was dominated by *Weissella* spp., but this dominance was not influenced by vegetable × level × probiotic (*P* > 0.05). Moreover, the RA of the *Acinetobacter, Stenotrophomonas, Prevotella, Leuconostoc*, *and Novosphingobium* genera was greater (*P* ≤ 0.03) in the pre-ensiled material inoculated with pumpkin (60% DM) and carrot (30 and 60% DM), as well as in uninoculated sorghum samples with carrot at 60% DM.

After 70 days of ensiling, the sample with 100% sorghum silage and probiotic inoculant and the silage samples supplemented with carrot or pumpkin at 30 or 60% DM, irrespective of inoculation with probiotics, were dominated by *Lactobacillus* spp. (Supplementary Table 2). Conversely, 100% sorghum silage without probiotic inoculation was dominated by the members of the *Weissella* genus (43.7 ± 3.66% RA), followed by *Pediococcus* (27.0 ± 2.32%), *Lactobacillus* (14.8 ± 1.41%), *Pantoea* (6.21 ± 1.41%), and *Klebsiella* spp. (3.26 ± 0.32%). Further, the effect of level × probiotic was observed on the RA of *Lactobacillus*, which was up to 84.7% lower in 100% sorghum without probiotic, compared to 30% vegetable with probiotic. *Pediococcus* spp. was 55.3% more relatively abundant (*P* < 0.01) in the 100% sorghum silage without the use of probiotics when compared to the silage treated with the probiotic.

The LEfSe tool was used to identify the bacterial and archaeal genera (Figure 2C), which likely contributed to the microbe-associated treatment differences observed in 70 day sorghum silage ensiled with carrot or pumpkin. Irrespective of the inclusion level, *Lactobacillus* spp. [log linear discriminant analysis (LDA) score = 5.53; *P* < 0.01] were enriched by the addition of carrots, while pumpkin treatments enriched *Acinetobacter* (log LDA score = 4.12; *P* < 0.01) and *Leuconostoc* spp. (log LDA score = 3.77; *P* < 0.01). The control silage had the greatest RA of *Bacillus* (log LDA score = 4.15; *P* = 0.02), *Klebsiella* (log LDA score = 4.24; *P* < 0.01), *Pantoea* (log LDA score = 4.49; *P* < 0.01), *Pediococcus* (log LDA score = 5.00; *P* < 0.01), and *Weissella* spp. (log LDA score = 5.22).

#### Pre-ensiled and Ensiled Community Structures

**FIGURE 3:**
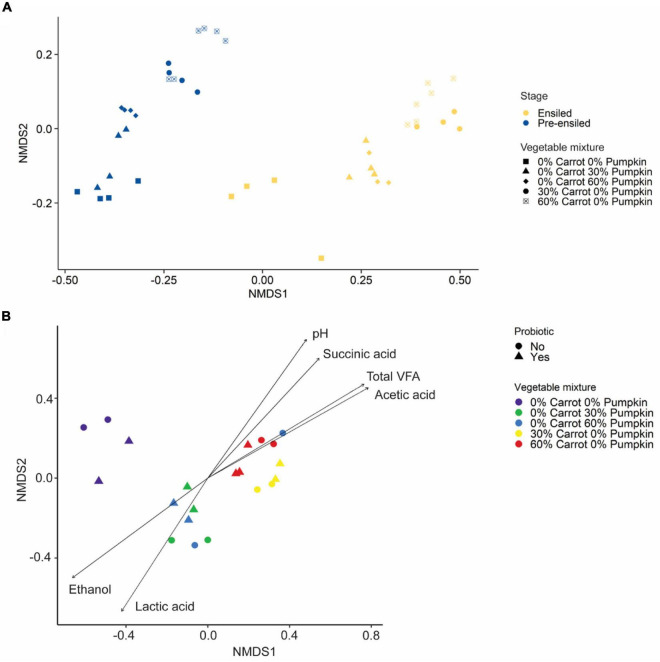
**(A)** Non-metric multidimensional scaling analysis (NMDS) based on the Bray–Curtis distance measure of pre-ensiled and ensiled sorghum with carrot or pumpkin at 0, 30, or 60% DM. **(B)** Non-metric multidimensional scaling analysis (NMDS) based on the Bray–Curtis distance measure of sorghum ensiled with either carrot or pumpkin at 0, 30, or 60% DM, with or without a probiotic inoculant. In **(B)**, vectors having a statistically significant association (*P* < 0.05) with the ordinations are included. Vector length is proportional to the degree of correlation between the fermentation parameters and the ordination.

The structure of the silage microbiota was strongly affected by the vegetable inclusion, irrespective of the type (PERMANOVA: *R*^2^ = 0.75; *P* < 0.001; Figure 3A), and less affected by the probiotic application (PERMANOVA: *R*^2^ = 0.08; *P* < 0.001). Further, the microbial community structure of silages containing carrots was significantly correlated with acetic acid (*R*^2^ = 0.82; *P* < 0.001; Figure 3B), succinic acid (*R*^2^ = 0.66; *P* < 0.001), and total VFA (*R*^2^ = 0.81; *P* < 0.001) concentrations and pH value (*R*^2^ = 0.72; *P* < 0.001). In contrast, the microbial community structure of silages containing pumpkin was significantly correlated with lactic acid (*R*^2^ = 0.62; *P* < 0.001) and ethanol (*R*^2^ = 0.69; *P* < 0.001) concentrations.

### *In vitro* Fermentation, Gas and CH_4_ Production, and Volatile Fatty Acids

There was no effect of vegetable × level × probiotic, vegetable × probiotic, or level × probiotic (*P* ≥ 0.08; data not presented) on any of the measured gas, CH_4_ production, or VFAs percentage (of total VFA), except percentage of caproic acid of total VFAs was influenced by vegetable level and probiotic addition. The results were separated on the basis of probiotic application.

#### No Probiotics

The interaction between vegetable type and level was significant (*P* ≤ 0.05; Table 5) for gas (mL, mL/g DM) production, *in vitro* dry matter digestibility (IVDMD), total VFA concentration, and branched-chain VFAs (BCVFA) (% of total VFA). Total gas production (mL) increased by 16.2 and 12.8% with the inclusion of carrot or pumpkin at 60% DM, respectively (*P* < 0.01; Table 5). Inclusion of carrot at 30% DM in uninoculated silages increased gas production (on an mL/g DM basis) by up to 16.2 and 14.2%, while the inclusion of pumpkin at 60% DM increased by 15.8 and 13.2%, respectively (*P* < 0.01). Additionally, gas produced per mL/g digestible DM was greater (*P* = 0.01) without probiotics in carrot rather than in pumpkin treatments, while increased production was also observed with the vegetable at 0% DM (*P* ≤ 0.01). The IVDMD was greatest with carrot or pumpkin at 60% DM in the uninoculated silages (45.1 vs. 48.7 ± 0.73%). Total VFA concentration and percentage of branched-chain VFA of total VFA were influenced by the interaction between vegetable type and level (*P* ≤ 0.01; Table 5), where total VFA was the greatest and BCVFA was reduced with 60% carrot or 30 and 60% pumpkin. No other effect of vegetable × level was observed on the *in vitro* VFAs (*P* ≥ 0.07). However, the inclusion of pumpkin to uninoculated silage increased percentage of caproic acid of total VFA from 0.34 to 0.42 ± 0.04 (*P* < 0.01).

**TABLE 5:** *In vitro* fermentation, gas production, and CH4 parameters of sorghum ensiled with carrot or pumpkin at 0, 30, or 60% DM without (no) probiotics.

No probiotic		Carrot		Pumpkin			*P*-values				
	0	30	60	30	60	SEM	Veg	Level	Veg × Level	Linear	Quadratic
Gas, mL	29.6b	29.3b	34.4a	33.8a	33.4a	0.75	0.06	<0.01	<0.01	<0.01	0.71
Gas, mL/g DM	63.8b	63.0b	73.9a	72.2a	71.7a	1.60	0.08	<0.01	<0.01	<0.01	0.63
Gas, mL/g DDM	170.5	164.2	164.2	149.1	147.3	3.57	0.01	<0.01	0.06	<0.01	0.08
CH_4_, %	10.5	10.2	9.3	10.4	11.9	0.66	0.11	0.91	0.14	0.87	0.69
CH_4_, mL g DM incubated	6.7	6.5	6.8	7.5	8.6	0.46	0.03	0.11	0.16	0.04	0.61
CH_4_, mL/g DDM	17.9	16.9	15.2	15.4	17.6	1.08	0.76	0.22	0.28	0.18	0.34
IVDMD, %	37.4c	38.5c	45.1b	48.7a	48.7a	0.73	<0.01	<0.01	<0.01	<0.01	0.07
pH	6.8	6.9	6.8	6.8	6.8	0.04	0.36	0.45	0.74	0.17	0.88
Total VFA, mM	81.4b	81.7b	97.6a	99.5a	99.5a	2.82	<0.01	<0.01	0.01	<0.01	0.76
**Individual VFA as a percentage of total VFA (mmol/100 mmol)**											
Acetic acid (A)	63.4	62.9	61.9	60.7	60.8	1.08	0.08	0.03	0.33	0.01	0.33
Propionic acid (P)	22.3	22.8	25.1	25.3	25.0	1.20	0.26	0.02	0.22	0.01	0.61
Butyric acid	11.0	11.1	10.0	11.09	11.35	0.32	0.10	0.43	0.07	0.30	0.43
Branched-chain VFA	1.72a	1.80a	1.41b	1.35b	1.29b	0.08	<0.01	<0.01	<0.01	<0.01	0.36
Valeric acid	1.12	1.15	1.13	1.10	1.16	0.05	0.86	0.87	0.71	0.62	0.89
Caproic acid	0.34	0.32	0.38	0.37	0.45	0.04	0.01	<0.01	0.11	<0.01	0.03
Ratio A:P	2.9	2.9	2.5	2.4	2.4	0.25	0.21	0.08	0.28	0.03	0.92

*SEM, standard error of the mean; IVDMD, in vitro dry matter digestibility. P-values for Vegetable × Probiotic and Vegetable × Level × Probiotic ≥ 0.08.*

*^a–c^Means with different letters differ (P ≤ 0.05) between levels within individual vegetable type (i.e., interaction Veg × Level was split by vegetable).*

#### Yes Probiotics

There was no effect of vegetable × level on any *in vitro* gas production parameter or VFAs, except BCVFAs, which tended to decrease with up to 60% pumpkin from 1.68 to 1.30 ± 0.07 (*P* = 0.05; Table 6). However, single fixed effects of vegetable and level (*P* ≤ 0.03) were observed on gas production (mL/g DDM), CH_4_ production (mg/g DM incubated, mL/g DM), and percentages of acetic, propionic, and caproic acids of total VFAs. Vegetable inclusion at 30 or 60% DM led to increased (*P* ≤ 0.01) *in vitro* gas production, on both volume basis (mL) and concentration (mL/g DM) basis, while the use of vegetables at 30 or 60% DM resulted in lower CH_4_ gas (*P* ≤ 0.01) production on a concentration (mL/g digestible DM) basis.

**TABLE 6:** *In vitro* volatile fatty acid composition of sorghum silages ensiled with carrot or pumpkin at 0, 30, or 60% DM, with (yes) probiotics.

Yes probiotic		Carrot		Pumpkin			*P*-values				
	0	30	60	30	60	SEM	Veg	Level	Veg × Level	Linear	Quadratic
Gas, mL	30.5	34.8	33.3	37.2	35.4	0.80	0.07	<0.01	0.29	<0.01	<0.01
Gas, mL g DM	65.9	74.4	72.2	79.8	75.9	1.78	0.08	<0.01	0.29	<0.01	0.01
Gas, mL g DDM	173.7	163.7	165.6	158.6	154.6	4.39	0.17	0.01	0.45	0.01	0.18
CH_4_, %	10.4	9.17	9.23	11.4	11.6	1.15	0.08	0.99	0.34	0.96	0.89
CH_4_, mL g DM incubated	6.84	6.82	6.91	9.15	8.96	1.00	0.05	0.27	0.28	0.19	0.43
CH_4_, mL g DDM	17.9	15.4	15.5	18.1	18.2	1.96	0.22	0.71	0.62	0.51	0.68
IVDMD, %	38.0c	45.0b	43.7b	50.5a	49.2a	1.21	<0.01	<0.01	0.04	<0.01	<0.01
pH	6.9	6.8	6.8	6.8	6.8	0.04	0.21	0.27	0.65	0.10	0.70
Total VFA	83.4	94.9	93.5	99.0	99.4	3.70	0.20	<0.01	0.60	<0.01	0.02
**Individual VFA as a percentage of total VFA (mmol/100 mmol)**											
Acetic acid (A) percentages of total VFAs	63.1	59.6	61.3	59.1	58.7	0.95	0.09	<0.01	0.18	<0.01	<0.01
Propionic acid (P)	22.6	26.1	24.2	26.0	25.7	1.29	0.53	0.01	0.61	0.02	0.01
Butyric acid	11.3	11.2	11.8	12.0	12.7	0.64	0.29	0.29	0.74	0.13	0.73
Branched-chain VFA	1.68a	1.50b	1.31c	1.30b	1.30b	0.07	0.06	<0.01	0.05	<0.01	0.02
Valeric acid	1.08	1.18	1.02	1.13	1.19	0.05	0.37	0.35	0.12	0.61	0.17
Caproic acid	0.34	0.38	0.34	0.46	0.42	0.05	0.01	0.02	0.13	0.14	0.01
Ratio A:P	2.8	2.5	2.7	2.3	2.3	0.25	0.17	0.06	0.52	0.07	0.09

*SEM, standard error of the mean; VFAs, volatile fatty acids; BCVFAs, branch-chained volatile fatty acids (iso-butyrate and iso-valerate).*

*^a–c^Means with different letters differ (P ≤ 0.05) between levels within individual vegetable type (i.e., interaction Veg × Level was split by vegetable). P-values for interactions Veg × Prob, Level × Prob, and Veg × Level × Prob were ≥ 0.08.*

Moreover, CH_4_ measured as mg or mL/g incubated DM was greater when pumpkin was combined with probiotics than with carrot (*P* = 0.03). When carrot or pumpkin at 30% DM was utilized with the probiotic application, IVDMD was found to be the highest in the inoculated silage (45.0 vs. 50.5 ± 1.21%; Table 6), and total VFAs increased by up to 15.6% with 30 or 60% DM (*P* < 0.01; data not presented). Ensiling sorghum with up to 60% DM vegetables decreased acetic acid and increased propionic acid percentages of total VFAs by 4.9 and 15.9%, respectively (*P* ≤ 0.01). The percentage of caproic acid of total VFAs was found to be influenced by both vegetable (*P* = 0.01) and level (*P* = 0.02), which was 17% greater with pumpkin when compared to the carrot. In addition, caproic acid was 20.5% higher in 30% DM vegetable treatment, compared to the 0% DM vegetable treatment.

## Discussion

The production of high-quality silage is dependent on effective microbial fermentation (Elferink et al., 2000). To achieve this in plant material lower than 50% DM, an estimated 10^8^ lactic acid bacteria per gram of plant material is required to inhibit the growth of clostridial spoilage microbes (Muck, 1988; Li et al., 2020), which can be observed due to increased lactic acid production. Herein, the concentration of lactic acid, highest with pumpkin at 60% DM, successfully decreased the pH of all the silage treatments to the pH range (4.3–4.7 vs. ≤ 4.35 ± 0.10) observed in fermented grass silages (Kung et al., 2018). The high lactic acid concentration and low pH of 3.68 ± 0.05 observed in the 100% sorghum silage treatment may be explained by the dominance of *Weissella* spp. (43.7 ± 3.66% RA; log LDA score = 5.22), which are epiphytic, homofermentative, and lactic acid-producing cocci bacteria commonly detected on grasses, such as sorghum (Cai et al., 1998).

Upon ensiling forage sorghum, Nazar et al. (2020) observed dominance of *Lactobacillus* (63.8%), *Weissella* (17.1%), and *Leuconostoc* (10.2%) spp., after 60 days of ensiling, while *Weissella* (43.7%), *Pediococcus* (27.0%), and *Lactobacillus* (14.8%) dominated in our uninoculated sorghum silage after 70 days of ensiling. Prior reports have determined that *Weissella* spp., may be detected in the samples that are contaminated by soil (Yang et al., 2010), while heterofermentative *Pediococcus* spp. have also been detected in grasses (Stirling and Whittenbury, 1963; Lindgren et al., 1983). Further, the presence of varied combinations of lactic acid bacteria is expected, as *Weissella* and *Pediococcus* spp. share similar phylogenetic traits with a variety of lactic acid-producing genera, including *Lactobacillus* (Cai et al., 1999). Interestingly, *Pediococcus* are lactic acid-producing bacteria that are known to colonize silages at a greater rate than *Lactobacillus* and have greater stability under a wide range of temperatures (Porto et al., 2017; Alhaag et al., 2019). The dominance of *Weissella* and *Pediococcus* spp., in 100% sorghum silage was similar to the findings reported by Ni et al. (2018), which is likely due to a lower pre-ensiling pH (Bolsen et al., 1996; Zeng et al., 2020).

The inoculation of sorghum samples with the BioSoil commercial probiotic, largely composed of *Lactobacillus* spp., resulted in the dominance of *Lactobacillus* spp. (46.5 ± 2.56% RA) in the silage, as expected. Irrespective of the inclusion level or probiotic inoculation, the RA of *Lactobacillus* was found to be 99.3 ± 2.56% in the microbial community of 30 or 60% DM carrot treatments. Furthermore, this dominance was evident, as all the measures of silage alpha diversity were lower with carrot inclusion at 30% DM. These findings were confirmed by the studies of Gallagher et al. (2018), and similar findings were also observed in our study, where *Lactobacillus* dominated sorghum ensiled with carrot at 30% DM. This led to a decrease in the sample silage microbial diversity metrics and resulted in the production of 15.2 ± 0.69 mM acetic acid (15.2 ± 0.69 mM), a secondary metabolite of soluble sugar fermentation by heterofermentative lactic acid bacteria (Martínez-Anaya et al., 1994). In addition, the NMDS biplot in this study illustrated that the separation of the carrot silage microbial community from other silage treatments may be best explained (log LDA score = 5.53) by the dominance of *Lactobacillus* spp., production of acetic and succinic acids, increase in the total VFA concentration, and decrease in the silage pH. In this study, silage pH of carrot inclusion for 30 or 60% DM was 18.5% greater than that observed in the control sorghum silage, suggestive of increased degradation of lactic acid into acetic acid by the heterofermentative taxa such as *Lentilactobacillus* (formerly *Lactobacillus*) *buchneri* in acidic pH conditions, that is, pH < 5.8 (Oude Elferink et al., 2001).

Moreover, ensiling wet (<30% DM; Bosma, 1991) to extremely wet (<25% DM; Shaver, 2003) plant material has previously resulted in the primary production of acetic acid in the resultant silages (Seppälä et al., 2016; Fijałkowska et al., 2020). Consistent with our findings, the DM content in the vegetable silage ranged from 15 to 22 ± 1.20% in this study, likely resulting in the accumulation of acetic acid *via* heterofermentative species of the genus *Lactobacillus* (Lindgren and Dobrogosz, 1990). Moreover, acetic and lactic acid production has also been observed as a product of the fermentation of glucose-rich carrot juices dominated by taxa within the *Lactobacillus* and *Leuconostoc* genera (Wuyts et al., 2018). Although prior studies have demonstrated that, during anerobic fermentation of carrots where *Leuconostoc* spp., were dominant, acetic acid and ethanol production was inhibited but lactobacilli growth was promoted (Gardner et al., 2001).

A distinct separation in the microbial communities of ensiled and pre-ensiled sorghum samples was noted in this study, as the ensiling process creates a unique environment that is conducive to the proliferation of anerobic microbes (Li and Nishino, 2013), rather than strict aerobes that occur as epiphytic microbiota on the surface of the starting plant material (McDonald et al., 1991). In the pre-ensiled microbial community, members of the genera *Salmonella*, *Clostridium*, and *Escherichia* are commonly detected in the plant material that has been contaminated by soil (Queiroz et al., 2018). Typically, soil contamination is characterized by an ash content exceeding 9% DM for legume grasses (Hoffman, 2005), while pre-ensiled sorghum with carrot or pumpkin at 20 or 40% DM had an ash content of 7 ± 0.47% DM (Forwood et al., 2019). In this study, the pre-ensiled sorghum had an ash content of 11.9 ± 0.44% DM, while the highest ash content of 15.8 ± 0.35% DM was observed for 60% pumpkin combined with sorghum among all the treatments.

However, *Salmonella*, *Clostridium*, or *Escherichia* spp., were not detected in any of the pre-ensiled epiphytic communities in the present study, despite a greater number of OTUs and Shannon’s and inverse Simpson’s diversity indices for the inclusion of carrot and pumpkin at 30% DM and of pumpkin at 60% DM. This is likely due to the utilization of carrots processed with sodium hypochlorite, which has previously reduced pathogenic microbial load by up to 99% (Betts and Everis, 2005). In line with prior reports, *Klebsiella*, *Weissella*, *Pantoea*, and *Pseudomonas* spp., dominated the epiphytic population of all pre-ensiled treatments, irrespective of inoculation with probiotics (Hu et al., 2018; Keshri et al., 2019). Moreover, the reports of Kõiv et al. (2019) confirmed our findings, as the members of *Pseudomonas* genus have previously been known to constitute up to 40% of the carrot peel and pulp microbiome population, while *Pantoea* is found to be a core genus. Similarly, in our study, *Pseudomonas* sp. was more prevalent and relatively abundant in the treatments including carrots at 30% (20.8 ± 1.87% RA) or 60% (13.9 ± 4.13%) DM, despite there being no significant influence of treatment on the pre-ensiled material.

Conversely, the NMDS ordination plot of ensiled material illustrated that the divergence of the pumpkin silage microbial communities might be best explained by pH and concentrations of total VFA, succinic acid, and acetic acid. Interestingly, *Acinetobacter* spp., which are soil microbes that utilize ethanol as a source of carbon (Abbott, 1973), and *Leuconostoc* spp., which are heterofermentative microbes that produce lactic acid and ethanol or acetic acid as a product of carbohydrate metabolism (Kandler and Schleifer, 1980; Dellaglio et al., 1995), were responsible for the differences (log LDA score ≥ 3.77; *P* < 0.01) in the lactic acid concentrations in the ensiled pumpkin treatments (log LDA score ≥ 3.77; *P* < 0.01), and higher lactic acid concentrations were also noticed in the present study.

The highest lactic acid concentration was observed for pumpkin inclusion at 60% DM and was likely due to the increased production by *Leuconostoc* spp. (Cogan and Jordan, 1994) in the presence of a higher quantity of fermentable sugars and water-soluble carbohydrates in pumpkin (Halik et al., 2018). Consequently, several reports have indicated that the presence of high water-soluble carbohydrate content following the inclusion of pumpkin increases the rumen-simulated DM fermentation by 26.5% with pumpkin inclusion in sorghum silage at 40% DM (Forwood et al., 2021), effective degradability in an *in situ* system by 13.8% w pumpkin inclusion in sorghum silage at 40% DM (Forwood et al., 2020), and IVDMD by 21 and 15.6% with pumpkin inclusion at 30% DM and 40% DM, respectively (Crosby-Galván et al., 2018; Forwood et al., 2019).

Similarly, our study observed that the inclusion of pumpkin in silages increased *in vitro* DM digestibility by up to 31.3% compared to 100% DM sorghum silage, which may be due to the rapid fermentation of non-fibrous carbohydrates in pumpkin during fermentation (Valdez-Arjona and Ramírez-Mella, 2019). Consequently, the inclusion of pumpkin at 60% DM yielded the greatest quantity of non-fibrous carbohydrates while maintaining the batch culture pH in the range of 6.79 to 6.85 ± 0.05, within the recommended pH range for rumen of 5.6–7.5 (Reis et al., 2014). This indicates that the presence of a suitable microbial environment with a sufficient quantity of substrates favors the fermentation process by rumen microbes. In addition, fermentation after 48 h of incubation led to increased gas production (mL/g), increasing by up to 17% with pumpkin inclusion at 30% DM, and corresponded with an increase in IVDMD, in contrast to a decrease noted in both the parameters with the use of pistachio by-products at up to 10% DM in a batch culture environment (Denek et al., 2017). Further, *in vitro* acetic and butyric acids were 14% lower and 31% greater with pumpkin inclusion, respectively, irrespective of level, and these findings are similar to those reported by Amer et al. (2012) and Chen et al. (2019).

## Conclusion

In conclusion, ensiling sorghum with unsalable carrot or pumpkin increases the total VFA silage concentration, indicating effective fermentation of plant material. This was subsequently proven by an increase in lactic acid concentration with carrot at 60% DM due to the dominance of *Lactobacillus* spp., in uninoculated vegetable silages, while the significant correlation between lactic acid concentration and the microbial community structure of the pumpkin silages at 60% DM indicated superior fermentation. This consequently promoted more digestible DM (e.g., increased silage IVDMD) and increased rumen fermentation in a batch culture incubation, as indicated by the higher concentration of total VFA. This study has provided further evidence supporting the use of unsalable carrots at 30% DM or pumpkin at 60% DM in silage as an alternative in silage production.

## Data Availability Statement

The datasets presented in this study can be found in online repositories. The names of the repository/repositories and accession number(s) can be found below: https://www.ncbi.nlm.nih.gov/sra, PRJNA699618.

## Ethics Statement

The animal study was reviewed and approved by The University of Queensland Production Animal Ethics Committee (approved protocol number: AE35581).

## Author Contributions

AC, DF, and SM conceived, designed research, and conducted experiments. DH and DF conducted bioinformatic analyses. AC, DH, DF, and SM analyzed the data and wrote the manuscript. AC, DH, and DF conducted statistical analyses. All authors read and approved the manuscript.

## Conflict of Interest

The authors declare that the research was conducted in the absence of any commercial or financial relationships that could be construed as a potential conflict of interest.

## Publisher’s Note

All claims expressed in this article are solely those of the authors and do not necessarily represent those of their affiliated organizations, or those of the publisher, the editors and the reviewers. Any product that may be evaluated in this article, or claim that may be made by its manufacturer, is not guaranteed or endorsed by the publisher.

## Footnotes

1
https://huttenhower.sph.harvard.edu/galaxy/

2
https://galaxyproject.org/
